# Multidimensional Health Impact of Multicomponent Exercise and Sustainable Healthy Diet Interventions in the Elderly (MED-E): Study Protocol

**DOI:** 10.3390/nu15030624

**Published:** 2023-01-25

**Authors:** Joana Sampaio, Joana Carvalho, Andreia Pizarro, Joana Pinto, André Moreira, Patrícia Padrão, Paula Guedes de Pinho, Pedro Moreira, Renata Barros

**Affiliations:** 1 Faculty of Sport (FADEUP), University of Porto, 4200-450 Porto, Portugal; 2 Research Centre in Physical Activity, Health, and Leisure (CIAFEL), University of Porto, 4200-450 Porto, Portugal; 3 Epidemiology Research Unit (EPIUnit), Public Health Institute (ISPUP), University of Porto, 4050-600 Porto, Portugal; 4 Laboratory for Integrative and Translational Research in Population Health (ITR), 4050-600 Porto, Portugal; 5 Associate Laboratory Institute for Health and Bioeconomy (i4HB), Laboratory of Toxicology, Department of Biological Sciences, Faculty of Pharmacy, University of Porto, 4050-313 Porto, Portugal; 6 Research Unit on Applied Molecular Biosciences (UCIBIO/REQUIMTE), Laboratory of Toxicology, Department of Biological Sciences, Faculty of Pharmacy, University of Porto, 4050-313 Porto, Portugal; 7 Faculty of Medicine (FMUP), University of Porto, 4200-319 Porto, Portugal; 8 Faculty of Nutrition and Food Sciences (FCNAUP), University of Porto, 4150-180 Porto, Portugal

**Keywords:** sustainable healthy diet, multicomponent training, metabolomics, health, elderly

## Abstract

Data concerning the combined effect of diet and exercise interventions on overall health in the elderly are scarce. The MED-E project’s primary aim is to assess the effect of the different 3-month sustainable healthy diet (SHD) and multicomponent training (MT) interventions on several health outcomes in the elderly. A quasi-experimental study assigned older adults into four groups: (1) SHD, (2) MT, (3) SHD + MT, or (4) control group (CG). The SHD intervention included a weekly offer of a mixed food supply and individual and group nutritional sessions on the principles of an SHD. The MT groups were submitted to 50-min exercise sessions three times a week. The primary outcomes were blood biomarkers and metabolic profile alterations that were assessed pre- and post-intervention. Additionally, data on dietary intake and nutritional adequacy, physical fitness, body composition and anthropometry, cognitive function, quality of life, and geographical data were assessed at the same time points. The MED-E project’s study protocol and future results will add to knowledge about the importance and beneficial contribution of combined SHD and MT interventions on healthy ageing policies.

## 1. Introduction

Population ageing is a concerning demographic phenomenon which is accompanied by a greater prevalence of chronic diseases. This disease burden increases healthcare costs and compromises individuals’ global health status. The adoption of lifestyle risk behaviours such as inadequate diets or under-activity may be a major contributor to poorer health statuses [1]. Studies report that unintentional nutritional and functional changes are more frequent in individuals aged 65 years or older [2,3].

Older adults have a dietary intake below recommendations in several nutrients: protein, n–3 fatty acids, dietary fibre, carotenoids, calcium, potassium, selenium, and vitamins B6, B12, D, and E [4]. It is also known that overconsuming saturated fat and sodium [4] is expected. The Mediterranean Diet [5] and the Dietary Approaches to Stop Hypertension (DASH) [6] are the most studied healthy dietary patterns, with more evidence regarding positive health benefits. These are characterised by high consumption of plant-based foods, e.g., vegetables, fruits, whole grains, and pulses, and low consumption of sweetened foods, refined grains, and processed meat [6]. Healthy, nutritionally balanced, and low environmental impact diets have been gaining awareness to improve population and planetary health [7,8]. Sustainable healthy diets (SHD) that are richer in plant-based and poorer in animal-based foods—such as the adapted Mediterranean Diet [5,9,10,11]—have been associated with nutritional adequacy; reduced body fat; decreased risk of weight gain, overweight, or obesity [8,12]; reduced frailty [13]; and better mental health [14]. Healthy dietary patterns improve lipidic (lowering triglycerides, total cholesterol, and low-density lipoprotein cholesterol—LDL-C) and inflammatory profiles, being associated with a reduction in C-reactive proteins (CRP), plasminogen activator inhibitor-1 (PAI-1), tumour necrosis factor-alpha (TNF-α), interleukin 6 (IL-6), and leptin [15,16].

Since older adults spend an average of 9.4 h/day engaged in a sedentary behaviours, which corresponds to 65–80% of their waking time [17], and are the least physically active segment of the population, engagement in physical activity (PA) routines should be considered in this age group. Exercise benefits to functional and cognitive capacities are well demonstrated in the literature [18], in addition to improvements in glycaemic control [19], inflammation [20], and body fat reduction [21]. Sampaio et al. (2019) demonstrated that regular multicomponent training (MT) sessions diminish the physiological effects of sedentary routines by improving quality of life, mobility, balance control, and bone mineral content [20], thereby delaying the development and progression of chronic and disabling conditions. Most studies regarding the impact of exercise on biomarker profiles were developed with different types of training (strength or aerobic programs). The literature shows a decrease in LDL-C, total cholesterol, CRP, IL-6, TNF-α, leptin, glycosylated haemoglobin A1c (HbA1C), insulin, and glucose, and an increase in high-density lipoprotein cholesterol (HDL-C) [22,23]. In MT programs, studies have corroborated previous findings concerning improving lipid profiles—lowering triglycerides and elevating HDL-C—and antioxidant capacity [24].

Moreover, studies regarding the combined effect of sustainable healthy dietary patterns and exercise interventions are scarce. The few existing studies suggest an intensification of the effects of isolated interventions with other beneficial health outcomes [25]. Besides the classical and standard biochemical analyses, untargeted nuclear magnetic resonance (NMR) spectroscopy-based metabolomics have begun to gain insight. This technique allows for the semi-quantification and analysis of hundreds of low molecular weight metabolites in biofluids, stools, and tissues [26,27], enabling the routine identification of novel compounds. Existing studies of diet and exercise approaches are based on short-term duration and strictly controlled and isolated diet or exercise intervention protocols, mostly with different population groups (such as children or younger adults) [28]. More extended intervention studies conducted under less strict diet and exercise programs are sparser. The qualitative and quantitative differences in metabolic profiles resulting from this combined intervention in the elderly have not yet been explored.

According to the socio-ecological model, an individual’s behaviour is affected by and affects the social environment. Therefore, holistic interventions are more practical in addressing healthy ageing [29]. Environmental factors may have an essential role in the modulation of food and physical activity (PA) habits [30]. Food, such as availability and accessibility to supermarkets, grocery or convenience food stores, and restaurants [30], and PA, such as details on walkability, parkland use, access to PA facilities [30,31], and environmental features must be considered. The use of Geographic Information Systems (GIS) can provide a novel and better understanding of the available environmental features, contributing to the implementation of effective healthy ageing policies [29].

Therefore, our primary aim is to assess the effect of different SHD and MT interventions on the biochemical and metabolic profiles, nutritional statuses and adequacy, physical fitness, body composition and anthropometry, cognitive function, and quality of life of older adults through a quasi-experimental study. Additionally, as a secondary aim, we intend to characterise food and PA environmental features and study their potential role in the modulation of food and PA practices.

## 2. Design and Methods

**Figure 1:**
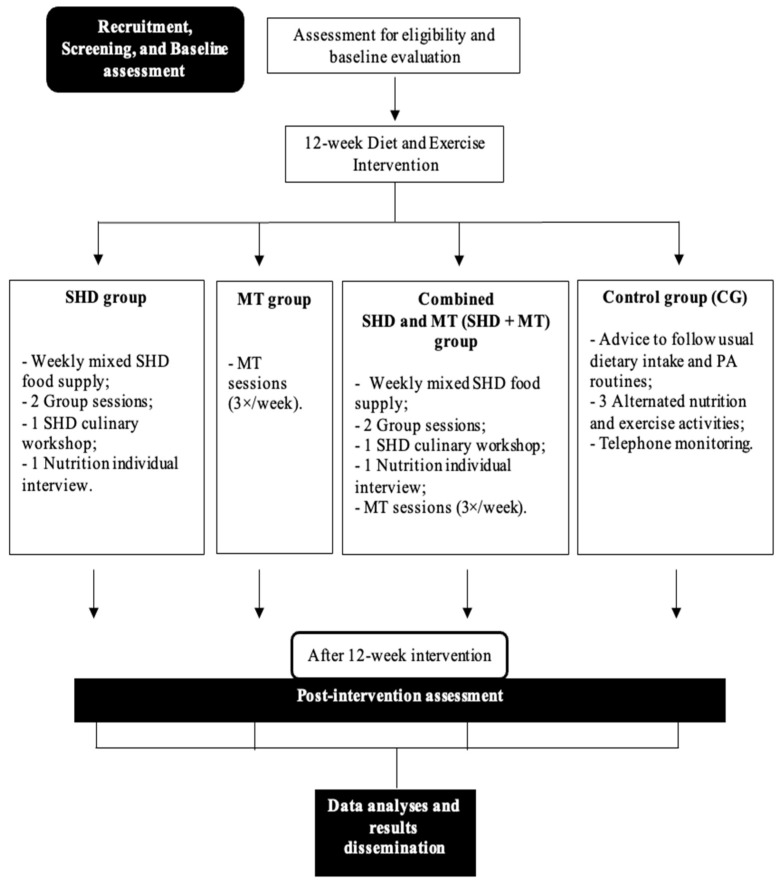
Protocol study flow-chart. SHD: Sustainable healthy diet; MT: Multicomponent training; PA: Physical activity.

The study was designed as a quasi-experimental, 12-week, four-armed, diet and exercise intervention with community-dwelling elderly residents from Porto, Portugal (Figure 1). This protocol report follows the Standard Protocol Items: Recommendations for Interventional Studies (SPIRIT) 2013 checklist [32]. 

### 2.1. Participant Involvement and Study Design

This multicentre quasi-experimental study was conducted in the Porto metropolitan area, Portugal. Participants were recruited from community programs, daycare centres, municipalities, healthcare centres, and social media platforms through phone calls, informative flyers, and presential and online meetings. Additionally, a short presential session was provided to motivate participation and clarify doubts about the program. Data collection started in November 2021 and ended in December 2022.

### 2.2. Eligibility and Recruitment

This study included individuals (i) aged 65 years or older, (ii) living in the community, (iii) with independent mobility, (iv) with no past medical history of neuropsychiatric conditions (e.g., major depression or bipolar disease), and (v) with no musculoskeletal or cardiovascular disorders that contraindicate participation in moderate exercise and testing. The exclusion criteria included individuals engaged in regular moderate to vigorous exercise training and followed by a nutritionist.

Eligible participants received a complete explanation of the study’s purposes, risks, and procedures. Before data collection, the principal researcher obtained informed consent from interested participants. After the agreement, participants were assigned into a 3-month SHD, MT, combined SHD and MT (SHD + MT), or control group (CG). 

Participants were assessed at baseline and after 3 months of intervention. At baseline, sociodemographic and economic, lifestyle (e.g., tobacco use and habitual physical activity), and general clinical data were collected through a structured questionnaire. Possible changes in these data were assessed in post-intervention assessments. At these two time points, nutritional and food intake evaluations were obtained through multiple reliable tools [33,34,35]: the Mini Nutritional Assessment [36]; physical capacity assessments through the Senior Fitness Test [37] and handgrip strength; a whole-body Dual X-ray absorptiometry (QDR 4500/A, Hologic Explorer, version 12.4, Bedford, MA, USA); a Montreal Cognitive Assessment [38]; the Quality of Life for Old Module (WHOQOL-OLD) [39]; and accelerometry (ActiGraph GT9X Link, Manufacturing Technology, Inc., South Bend, IN, USA) were performed. 

Participants were only informed about which group they belonged to after baseline assessments. Well-trained and skilled researchers executed the same procedures during the two assessment periods.

### 2.3. Sample Size Estimation

The sample size was estimated based on previous literature and the ANCOVA to analyse the differences between and within groups [40]. A sample size calculation was conducted using G*Power 3.1.3 (Universität Düsseldorf, Düsseldorf, Germany) [41]. We predicted the inclusion of 25 participants per group, a total of at least 100 community-dwelling older adults, both men and women, considering a moderate effect size of 0.40, an alpha-risk of 0.05, and a beta-risk of 0.20 in a 2-sided test, and anticipating a 20% dropout rate.

### 2.4. Ethics Approval and Data Management 

The study protocol was approved by the Ethical Committee of the Faculty of Food and Nutrition Sciences of the University of Porto (reference 17/2021/CEFCNAUP/2021), the Ethics Committee of Northern Region Health Administration (report CE/2022/71), and the Data Protection Unit of the University of Porto. The project procedures followed all the ethical standards and the 1964 Helsinki Declaration. 

Before data collection, the principal researcher obtained informed consent from interested participants. 

Data coding, security, and storage procedures followed the guidelines of the Data Protection Unit of the University of Porto. Information regarding confidentiality and the transfer of data obtained within the scope of the MED-E project is present in the study information document for participants, attached to the informed consent. Data confidentiality and anonymity were guaranteed in all phases of the study.

### 2.5. Study Intervention

#### 2.5.1. Sustainable Healthy Diet Group (SHD) 

Diet intervention lasted approximately three months. Participants were provided, weekly, with an SHD food supply, including planted-based foods (nuts and pulses), olive oil, and oily fish, which might have contributed to higher compliance with the overall dietary pattern. Based on the Mediterranean Diet and the latest EAT-Lancet recommendations [12], the individual food supply included per week 125 g of nuts (walnuts, considering a daily serving of 25 g); 350 g of dry pulses (chickpeas and different types of beans, based on a daily serving of 50 g); 280 g of olive oil (a bottle of olive oil was delivered per month); and two servings of oily fish (sardine fillets and tuna, 100 g servings). 

Additionally, the intervention included a total of four sessions: three group sessions (including an SHD culinary workshop) and one telephone interview ranging from 30 (the individual session) to 60 min with a trained nutritionist at the beginning, 1-month, 2-months, and 2.5-months into the intervention. The group and individual sessions were held by nutritionists. The focus was on acquiring knowledge and maximising individual participation. Group sessions involved a maximum of 25 participants. All the sessions were held emphasising participants’ involvement to allow for more dynamic and interactive meetings.

The first group session was an introductory one explaining the principles of an SHD and the benefits of this pattern in several health outcomes. The participants learned how to evaluate environmental impact, and some examples of food footprints were given. The recommended serving sizes of an SHD and the seasonal foods for each month were also presented. 

The second group session was an SHD culinary workshop in an experimental kitchen laboratory of Faculty of Nutrition and Food Sciences (FCNAUP) to increase participants’ knowledge, skills, and literacy regarding SHD recipes. In this SHD culinary workshop, seasonal and Mediterranean-based local food products were chosen. Participants were organised into four groups, and each was required to elaborate on recipes. The participants tasted all the recipes at the end of the SHD culinary workshop. The workshop included as recipes (i) a soup with a traditional and local Portuguese green kale named Caldo Verde; (ii) a cold Mediterranean mackerel salad with pulses; (iii) roasted mackerel with potatoes and seasonal vegetables (onion, garlic, different colour peppers, broccoli, and carrots); (iv) and sardines rice with turnips and greens. Dessert included seasonal fresh fruits and walnuts.

The third group session included tips on supermarket shopping and food storage and a novel approach to tackling food waste to capacitate the participants to choose fresher, healthier, traditional, seasonal, and cheap products. Participants learned how to promote sustainability during culinary activities. Sustainable and healthy weekly meal plans and cooking recipes were provided. 

In individual interviews, food and nutritional education considered baseline dietary intake and nutritional adequacy assessments, and recommendations in line with the latest EAT-Lancet recommendations for adopting an SHD were set [12].

#### 2.5.2. Multicomponent Training Group (MT) 

The MT exercise program included 50-min group sessions three times a week on non-consecutive days. The sessions were distributed into three main parts: 8–10 min of warm-up (including walking, postural, mobility, and stretching exercises), 30–35 min of specific training (including balance, coordination, strength, and aerobic conditioning), and 5 min cooldown (breathing and stretching exercises). Exercise prescription followed the main guidelines from the American College of Sports Medicine [42] and the World Health Organization (WHO) [43]—60–80% of maximum heart rate (HRmax) and 50–70% of one-repetition maximum (1RM). 

Simple, age-adapted, enjoyable, and functional exercises were preferred. Before the training program, a two-month adaptation period was implemented to familiarise the participants with the activities, observe their individual physical needs and promote socialisation among them. The adaptation period maintained a low-intensity range of 40 to 50% HRmax in the aerobic exercises and one set of 12–15 slow repetitions for the strength exercises. The focus was on learning the movements. Exercise intervention prescription, implementation, and evaluation were performed by elderly specialist exercise professionals. Sessions involving a maximum of 25 participants were held in appropriate, safe gymnasiums. Whenever possible, sessions were accompanied by music to promote participants’ involvement and enthusiasm and confer intensity. Balance exercises gradually reduced the base of support and/or reduced sensory input, and included dynamic movements that perturbed the centre of gravity. Additional focus was placed on some easy coordination exercises and conscious control of the body. Four to six multi-joint strength exercises involving major muscle groups were included in each session. The number of repetitions gradually decreased (12–15 to 10–12), but the load increased and could be lifted correctly to volitional fatigue. A rest period was completed between sets. Aerobic endurance was achieved with low-impact exercises, gradually increasing intensity, duration, and HR. The Rated Perceived Exertion (RPE) scale was used to support the exercise intensity control—as medication induces HR alterations and adverse events (such as pain or fatigue). The number of falls was recorded at the end of each MT session. Session attendance was recorded [44] and participants missing more than 12 consecutive sessions (equivalent to one month of detraining) were excluded from the final analyses.

#### 2.5.3. Combined Sustainable Healthy Diet and Multicomponent Training Group (SHD + MT) 

In this group, participants received the same SHD and MT interventions as the participants of the abovementioned groups: a weekly sustainable and healthy mixed food supply and the additional three group sessions (including the SHD culinary workshop) and one individual telephone interview, in addition to MT 50-min group sessions three times a week on non-consecutive days. 

#### 2.5.4. Control Group (CG)

The CG received advice on following habitual dietary and PA routines. During the project intervention period, one nutrition session regarding the main principles of a healthy diet and two low-intensity PA sessions were offered to the participants in this group. Weekly telephone contact was conducted to ensure participant retention in the study.

### 2.6. Outcome Assessments

#### 2.6.1. Primary Outcome

##### Blood-Based Biomarkers and Metabolic Alterations

Our primary outcomes concern blood-based biomarkers and metabolic alterations. At baseline and post-intervention, venous blood samples were taken. All the biochemical and metabolomic analyses—untargeted NMR—were performed in a certified laboratory. 

After an overnight fast of at least 8 h, a competent professional collected venous blood samples from the antecubital vein (required volume between 10 and 20 mL, maximum of two tubes) in a specially designated and equipped room, separating samples into serum and plasma. The following nutritional and age-related biomarkers [45] will be analysed in serum blood samples: glucose; fructosamine; insulin; cortisol; protein C-reactive (high sensitivity); vitamin B12; iron; sodium; phosphorus; calcium; magnesium; lipid profiles: total cholesterol, HDL-C, LDL-C, and triglycerides; and osmolality. Traditional biochemical parameters will be enzymatically measured [46]. LDL-C will be computed according to the Friedewald formula, estimated by subtracting HDL-C and one-fifth of the triglycerides value from the total cholesterol level [47]. 

We also intend to perform untargeted NMR-based metabolomics analyses. Plasma samples were immediately frozen at −80 °C and will remain so until analyses are conducted. Blood plasma preparation for NMR analyses will include adding saline solution (sodium chloride (NaCl) 0.9%, 10% deuterium oxide) to plasma, followed by centrifugation and transfer to 5-mm NMR tubes. The NMR analyses will be performed in a Bruker Avance III HD 600 MHz spectrometer (Bruker BioSpin, Rheinstetten, Germany) equipped with a cryoprobe using a Carr–Purcell–Meiboom–Gill (CPMG or T2-edited) pulse sequence (cpmgpr1d) [48]. For one representative sample, 2D spectra (heteronuclear single quantum coherence (HSQC) and total correlation spectroscopy (TOCSY)) will be acquired to aid in metabolite annotation.

#### 2.6.2. Secondary Outcomes

##### Dietary Intake Assessment and Nutritional Adequacy

Dietary intake was assessed through a 24-h dietary recall method, including two non-consecutive days, following European Food Safety Authority recommendations [33]. A 24-h dietary recall is a structured interview that collects detailed information (time of day, food source, portion size, and preparation method) about all foods, beverages, and dietary supplements consumed by the participant in the past 24 h. A food model manual with pictures was used to help respondents judge and report portion size and improve accuracy. Information regarding nutritional supplements was also collected (such as type of supplement, name, and dosage).

Energy and nutritional intake, based on the 24 h recalled food data, were estimated using the nutritional analysis software Food Processor®, version SQL 11.11.32 (ESHA Research Inc., Salem, OR, USA).

Additionally, a Food Propensity Questionnaire [34] was applied to estimate usual and seasonal long-term dietary intake based on the preceding year. The frequency of consumption was based on the following food groups: (i) bread- and cereal-based foods, baked goods, and sugar; (ii) vegetables and fruits; (iii) nuts, seeds, and pulses; (iv) meat (including red and processed meat products); (v) fish (including oily-fish) and seafood; (vi) eggs; (vii) dairy products and substitutes; (viii) fruit juices and soft drinks; (ix) alcoholic beverages; and (x) coffee and tea. Seven potential response options were given to estimate the frequency of intake: never, <1 time per month, 1–3 times per month, one time per week, 2–3 times per week, 4–5 times per week, and 6–7 times per week.

The Mediterranean Diet Adherence Screener (MEDAS) Questionnaire [35], validated in the Portuguese population, was used to assess participants’ adherence to the Mediterranean Diet, a traditional gold model of an SHD. The total score of this questionnaire ranges between 1 and 14, in which scores greater than 9 indicate high adherence to the Mediterranean Diet [35,49]. 

Semi-structured questionnaires to measure compliance with the consumption of the food products in the food supply were applied every week. The consumption of different products from the same food groups was also assessed.

The nutritional status assessment was realised through the short form of Mini Nutritional Assessment (MNA-SF) [36], previously translated into Portuguese. This screening tool identifies participants that were at risk of malnutrition or malnourished. This tool comprises six questions, scored from zero to three. The maximum total score is 14 points. Participants scoring 0–7 points were considered malnourished, 8–11 points were at risk of malnutrition, and those above 12 points had a normal nutritional status.

##### Physical Fitness

Physical fitness was assessed by the Senior Fitness Test (SFT) [37], a trustworthy method for assessing physical capacity in the elderly that includes six functional tests: (i) the chair stand test, which reflects lower body strength, measured for 30 s; (ii) the arm curl test, to measure upper body strength for 30 s; (iii) the six-minute walk test, to assess aerobic endurance; (iv) the chair sit and reach test, to measure lower body flexibility; (v) the back scratch, that assesses the upper body flexibility; and (vi) the time up-and-go test. 

Static balance was also measured with the One Leg Balance Test [50]. In this test, the participant stood unassisted on one leg and the maximum time (45 s) reached, in seconds, from the moment one foot was off the floor to the moment it touched the other leg or the ground was registered. The participant was allowed three attempts, and the best result was recorded.

Finally, handgrip strength was obtained with a digital Jamar hand dynamometer (Sammons Preston Inc., Illinois, IL, USA). According to the American Society of Hand Therapists [51], individuals sat comfortably in a chair without armrests with the shoulder in adduction and neutral rotation, the elbow in 90° flexion, and the forearm and wrist in a neutral position, between 0° and 30° of dorsiflexion. In the handgrip strength test, participants performed three trials at one-minute intervals with each hand (information on the dominant hand was recorded). 

##### Body Composition and Anthropometry

Body composition was analysed through DXA (QDR 4500/A, Hologic Explorer, version 12.4, Bedford, MA, USA), a valid and reliable method in the elderly population for whole-body lean and fat mass, body fat percentage, and trunk fat, with subjects in the supine position.

Anthropometric measurements were taken using international standardised protocols. Body Mass Index (BMI) was determined to the nearest 0.1 kg with an electronic portable weight scale (Inbody 120, Seoul, Republic of Korea). Overweight and obesity will be defined using international criteria [52]. Waist and hip circumference were assessed using a spring-loaded measuring tape parallel to the floor. Waist circumference was measured at the midpoint between the iliac crest and the bottom of the ribcage and the hip circumference at the broadest portion of the buttocks [53].

##### Cognitive Function

The Montreal Cognitive Assessment (MoCA) [38,54], previously translated into Portuguese, a commonly used cognitive function test among older adults, was applied. It is a brief test, scored to a maximum of 30 points, with higher scores reflecting better cognitive performance. The MoCA assesses several cognitive domains. Visuospatial capabilities were evaluated through a clock-drawing task (3 points) and a 3D cube copy (1 point). Executive functions were assessed using a task adapted from the Trail Making B task (1 point), a phonemic fluency task (1 point), and a two-item verbal abstraction task (two points). The short-term memory recall task (5 points) involved two learning trials of five nouns and a delayed recall after approximately 5 min. Attention, concentration, and working memory were evaluated using a sustained attention task (1 point), a serial subtraction task (3 points), and digits forwards and backwards (1 point each). The language domain was measured using a three-item animal naming task (3 points) and the repetition of two syntactically complex sentences (2 points). Temporal and spatial orientation was evaluated to a total of 6 points.

##### Quality of Life

The Quality of Life for Old Module (WHOQOL-OLD) [39] was used to measure the participants’ quality of life. The WHOQOL-OLD module consists of 24 items categorised into the following six domains, answered on a 1–5 Likert scale: sensory abilities; autonomy; past, present, and future activities; social participation; death and dying; and intimacy. Higher scores reflect a greater quality of life.

##### Geographical Data

Geographical data will be gathered using several data sources and systematised in a geodatabase in ArcGIS, version 10.5 (Environmental Systems Research Institute, California, USA). The variables’ selection criteria were based on previous literature and included walkability and access to green spaces, fast-food restaurants, grocery and convenience stores, and supermarkets. To measure pedestrian access, we will use a 400-m street-network buffer as a reasonable walking distance to neighbourhood resources.

#### 2.6.3. Confounders

As possible confounders, we will control at baseline and after the intervention for (i) sociodemographic and economic status, (ii) clinical history, (iii) tobacco use, (iv) medication (collected through a structured questionnaire), and (v) daily physical activity, assessed through an accelerometry-based method (ActiGraph GT9X Link, Manufacturing Technology, Inc., South Bend, IN, USA). Participants were instructed to always wear the accelerometer for seven consecutive days attached to an elastic belt, except when sleeping or during water activities. Data were collected in 100 Hz epochs; valid data must include a minimum of 10 h on at least four days (with one weekend day). This data will be processed using Actilife™ software, version v6.13.4 (Actigraph LLC, Pensacola, FL, USA) and organised as sedentary, light, moderate, and vigorous physical activity.

### 2.7. Statistical Analyses

Descriptive results will be expressed as median and inter-quartile range (IQR) or mean and standard deviation (SD), as appropriate, for continuous variables. Discrete variables will be presented as counts and percentages. The minimum threshold value for statistical significance used will be *p* ≤ 0.05.

The normality tests, histogram, and plot distribution will assess the normality of data distribution. Comparative analyses between participants and dropouts will be realised by *t*-test or Mann–Whitney U-test for continuous variables and Pearson-Chi Squared or Fisher exact test for categorical variables to determine whether attrition bias was observed. 

Potential differences among intervention groups in baseline measurements will be evaluated using a one-way Analysis of Variance (ANOVA) or correspondent non-parametric Kruskal-Wallis test. A Pearson Chi-squared or Fisher exact test will be used for between-group comparisons of categorical variables. 

Covariates (e.g., sociodemographic and economic characteristics; clinical history, such as the presence of comorbidities; and daily physical activity) were selected a priori and tested. The confounding variables will be entered as covariates in the ANOVA models. For each model, the contribution of all covariates will be tested separately; only the ones that change the magnitude of the association by at least 10% will be included in the final models. The fit of the models will be determined. Whenever necessary, we will use weighted samples to tackle the bias imposed by differential sampling probabilities.

A delta percentage will be calculated to assess the percentage of change between baseline and post-intervention assessments. An ANOVA or correspondent non-parametric test with repeated measures will be performed for differences in main effects and interactions for each dependent variable. When significant interactions are found, Bonferroni posthoc tests will determine the origin of the differences. Multivariable regression analyses will explore the associations between blood-based markers and metabolomics and the secondary outcomes.

Geographical information systems data will be collected. The associations of the primary and secondary outcomes with environmental features will be explored through generalised linear models to understand whether built environment food and PA features lead to statistically significant differences in participants’ dietary and exercise routines.

Multivariate analyses will be applied to discriminate between the metabolic profiles of the intervention groups, enabling the identification of the most relevant panel of spectral peaks differing between them [55]. Then, univariate statistical analyses, including hypothesis testing (ANOVA or the corresponding non-parametric test), effect size, and the area under the curve (AUC) obtained by receiver operating characteristic (ROC) analyses will be applied to test the statistical relevance of the panel of spectral peaks [55]. The metabolite annotation in NMR data will be performed. A comparison with the NMR spectral profiles of standard compounds presented in databases and the validation in 2D spectra (heteronuclear single-quantum coherence (HSQC) and total correlation spectroscopy (TOCSY)) acquired for one representative sample will be completed. In addition, the metabolic pathways altered in the groups under study will be interpreted through metabolomic pathway analyses [56].

All statistical analyses performed using the SPSS IBM Statistical Software version 25.0 (SPSS, Inc., Chicago, IL, USA) for macOS (Apple Inc., Cupertino, CA, USA) will be used for data assembly and graphical analyses.

## 3. Discussions

We expect to disseminate study results by publishing manuscripts in peer-reviewed scientific journals and giving presentations at national and international conferences. Additionally, we will promote seminars for the entire community, particularly for the participants and healthcare organisations, and disseminate essential results through institutional public media (webpage, social media networks). Participants will be informed individually of their study results by synthesised reports.

This study describes the protocol of a quasi-experimental study of 3-month SHD and MT interventions for older adults comprehended on the MED-E project. 

Some innovative aspects of this study should be underlined. Data were obtained from an urban population of a high-income South European country living in Porto, mostly from Paranhos (same parish), with similar baseline conditions regarding physical activity and sociodemographic and economic conditions, which empowers the study. Most outcome variables were objectively assessed, reducing the risk of measurement or misclassification errors. Additionally, having the same intervention location for all participants will minimise bias. Moreover, this project followed the latest recommendations for diet and exercise interventions for this population group. Using cutting-edge technologies, specifically NMR-based metabolomics, we aim to determine how diet and exercise can impact several biomarkers and metabolites. Compared to most studies on the impact of nutritional and dietary interventions on health, this study includes an SHD food intervention. It also has a more extended intervention period than is usually observed. It has a practical component, promoting the direct involvement of participants in the SHD culinary workshop. Additionally, continued, individually adjusted, and personalised MT sessions may positively impact the functionality and cognitive capacity of older adults and improve their quality of life. Our findings will provide needed insights and help to strengthen the current evidence regarding nutrition and exercise as complementary adjuvant approaches to promoting healthy and active ageing.

Some challenges to this study must be recognised. Firstly, we anticipate a potential sample with several age-related comorbidities. Secondly, the elderly tend to mainly adopt sedentary behaviours, increasing difficulties in the recruitment process. Based on differences in baseline participant characteristics, a misinterpretation of the primary outcomes and an inability to generalise the results may occur. Considering the main inclusion criteria, the different interventions cannot be generalised to other specific populations. Despite including participants with lower nutritional status or who were inactive, we cannot exclude the possibility that the participants involved in the study were more prone to healthy lifestyle behaviours. Though, this bias was minimised by excluding participants that were engaged in regular physical activity or consulting a nutritionist. Additional research will help identify the most effective diet and exercise intervention protocols for more heterogeneous, non-uniform, and other specific population groups. A cross-over and randomised controlled trial (RCT) design, where the same participant implemented different interventions, would reduce the likelihood of heterogeneity affecting the evaluation of primary outcomes. Initially, our study protocol was thought and designed to be a cross-over RCT. Although, the methodology needed to be adapted due to the beginning of the unpredictable pandemic situation, which occurred simultaneously with the start of the recruitment study and disabled the sample randomisation. The pandemic situation delayed and imposed logistical difficulties in the recruitment process. The lockdown and isolation of this at-risk population group (older adults) affected baseline assessments and the study intervention due to the closure of facilities.

## 4. Conclusions

The MED-E project’s study protocol and future results will add to knowledge about the importance and beneficial contribution of combined SHD and MT interventions on healthy ageing policies, promoting an improvement in age-related health indicators. Moreover, the study purposes align with the World Health Organization’s recommendations for the functional health era and the United Nations’ 2030 goals. This study protocol promotes good health and well-being with the implementation of holistic active and healthy lifestyle interventions. Furthermore, nutritional intervention provides participants with knowledge regarding sustainable diets, cities, and communities, and the weekly food supply supports and promotes the national economy and local production. 

## Data Availability

Data sharing does not apply to this article as no datasets were generated or analysed during the current study.
